# Lingering expectations: A pseudo-repetition effect for words previously expected but not presented

**DOI:** 10.1016/j.neuroimage.2018.08.023

**Published:** 2018-08-11

**Authors:** Joost Rommers, Kara D. Federmeier

**Affiliations:** a Department of Psychology, University of Illinois, Urbana-Champaign, USA; b Beckman Institute for Advanced Science and Technology, University of Illinois, Urbana-Champaign, USA; c Donders Institute for Brain, Cognition and Behaviour, Centre for Cognitive Neuroimaging, Radboud University Nijmegen, The Netherlands; d Program in Neuroscience, University of Illinois, Urbana-Champaign, USA

**Keywords:** Prediction, Language comprehension, Word repetition, Event-related potentials (ERPs), N400, Alpha

## Abstract

Prediction can help support rapid language processing. However, it is unclear whether prediction has downstream consequences, beyond processing in the moment. In particular, when a prediction is disconfirmed, does it linger, or is it suppressed? This study manipulated whether words were actually seen or were only expected, and probed their fate in memory by presenting the words (again) a few sentences later. If disconfirmed predictions linger, subsequent processing of the previously expected (but never presented) word should be similar to actual word repetition. At initial presentation, electrophysiological signatures of prediction disconfirmation demonstrated that participants had formed expectations. Further downstream, relative to unseen words, repeated words elicited a strong N400 decrease, an enhanced late positive complex (LPC), and late alpha band power decreases. Critically, like repeated words, words previously expected but not presented also attenuated the N400. This “pseudo-repetition effect” suggests that disconfirmed predictions can linger at some stages of processing, and demonstrates that prediction has downstream consequences beyond rapid on-line processing.

## 1. Introduction

The brain has been argued to be a prediction machine that continuously compares sensory input against internally generated expectations (Bar, 2007; Clark, 2013). As long as the expectations are confirmed, this may facilitate stimulus processing; when expectations are instead disconfirmed, the resulting error signals are thought to promote learning (Friston, 2005; Rao and Ballard, 1999). In language comprehension studies, scalp-recorded event-related brain potentials (ERPs) have revealed that word predictability reduces the amplitude of the N400, a centroparietally distributed negativity that peaks around 400 ms after stimulus onset and has been associated with semantic processing (Kutas and Hillyard, 1980, 1984; for review, see Kutas and Federmeier, 2011). Unexpected but plausible words read in sentences wherein they disconfirm a likely prediction elicit a later, frontally distributed positivity (Federmeier et al., 2007; see also DeLong et al., 2014; Van Petten and Luka, 2012). In addition to ERPs, which highlight brain activity phase-locked to stimulus onset, time-frequency analyses of power provide a window into non-phase-locked (often oscillatory) activity, which is thought to reflect rhythmic fluctuations in excitability useful for communication between brain areas (e.g., Fries, 2005). Relative to predictable words, unexpected words elicit power increases in the theta band (4–7 Hz; e.g., Hald et al., 2006; Rommers et al., 2017; Wang et al., 2012).^1^ However, beyond processing in the moment, extant electrophysiological data leave open the question of whether prediction disconfirmation has any downstream consequences for the representations that comprehenders ultimately retain.

In particular, it is unclear what happens to an expectation after it has been disconfirmed. Is it suppressed, or does it linger? On the one hand, suppression of the originally expected representation by a revision process seems important for obtaining an accurate interpretation of the input. Indeed, one hypothesized functional correlate of the frontal positivity mentioned above is this kind of suppression or inhibition, which may also be relevant for learning (Federmeier et al., 2007; Kutas, 1993; for a learning framework, see Chang et al., 2006). On the other hand, disconfirmed expectations might linger if their downstream consequences are similar to those of temporarily ambiguous input. It has been shown, for example, that after reading a garden-path sentence such as “While Anna dressed the baby spit up on the bed”, readers often incorrectly believe that Anna dressed the baby (Christianson et al., 2001; see also Kaschak and Glenberg, 2004; Slattery et al., 2013; for review, see Ferreira and Patson, 2007). This suggests that, at least for actually presented input, reanalysis can be incomplete, and temporarily possible interpretations can persist.

A few previous studies, aimed at questions beyond basic comprehension, have reported memory performance for expected but not presented words. After intentional encoding of words in sentences, a subsequent sentence completion task showed lingering of previously disconfirmed expectations in older adults; however, younger adults did not show this effect, perhaps because they were better able to suppress previously relevant information (Hartman and Hasher, 1991). Younger adults have shown lingering in the form of false alarms in recognition memory, in a study in which predictable spoken words were not disconfirmed by an unexpected alternative, but instead replaced by silence (Foucart et al., 2015).^2^ Importantly, lingering or suppression may be reflected in one or multiple specific processes over time, which end-state measures of memory performance summate across.

The present study examined the fate of disconfirmed expectations using the EEG signal elicited by incidental repetitions during reading for comprehension. Specifically, this study manipulated whether words were actually seen or were only expected, and probed their fate in memory by presenting the words (again) a few sentences later. If disconfirmed expectations linger, subsequent presentation of the previously expected but not presented word should be similar to actual word repetition. If disconfirmed expectations are suppressed, presenting the previously expected word should not be similar to repetition.

The repetition effect is multifaceted and has been well characterized in ERP studies. Relative to initial presentation, repeated words typically elicit a positivity consisting of a reduced N400 (Rugg, 1985; Van Petten et al., 1991) and an enhanced late positive complex (LPC; Besson et al., 1992; Rugg et al., 1998). The LPC has been taken to index recollection, because it is enhanced when recognition is part of the task (Paller and Gross, 1998), after deep encoding tasks that yield strong recollection (Paller and Kutas, 1992; Rugg et al., 1998), when words are explicitly recognized as old (Van Petten and Senkfor, 1996), and when memories include episodic details pertaining to encoding modality or source (Wilding and Rugg, 1996; Wilding et al., 1995). In contrast, the N400 decrease has been associated with more implicit priming processes, because it can occur relatively independently of the depth of memory encoding (Paller and Kutas, 1992) or recognition memory accuracy (Rugg et al., 1998). Strikingly, in patients with amnesia, known for intact implicit memory but impaired explicit memory abilities, the N400 repetition effect is preserved but the LPC is not (Olichney et al., 2000).

In addition to effects seen with ERPs, word repetition also results in power decreases in the alpha band (8–12 Hz) of the EEG after 500 ms post-stimulus, an effect seen in both word lists (Van Strien et al., 2007) and sentences (Rommers and Federmeier, 2018). Such alpha decreases have been linked with the re-activation of memory traces (Klimesch et al., 2005). During retrieval tasks, alpha decreases can vary in topography with the type of studied material (Burgess and Gruzelier, 2000; Khader and Rösler, 2011), and the decrease is stronger when more as opposed to fewer items need to be retrieved (Khader and Rösler, 2011), as well as after retrieval practice compared with merely studying items (Spitzer et al., 2008). Taken together, the electrophysiological nature of observed repetition effects can shed light on the memory processes affected by a manipulation.

Against this background, the present study examined the fate of disconfirmed expectations using the N400, the LPC, and (alpha) power. Participants read weakly constraining sentence contexts ending in a critical word (“hot”). The conditions differed with respect to what had been presented previously. The critical word had been presented a few sentences earlier, had not been presented, or had only been expected before being disconfirmed by a plausible alternative (“Be careful, because the top of the stove is very dirty”, where “hot” was expected). An example is shown in Table 1. Relative to previously unseen words, repeated words were expected to elicit an N400 decrease, an LPC increase, and late power decreases in the alpha band. Critically, if disconfirmed expectations linger, similar effects may be observed in response to the previously expected but not presented words. If, instead, disconfirmed expectations are suppressed, previously expected but not presented words should not elicit repetition-like effects – and in the event that previously expected information is not only suppressed but even inhibited, these words could elicit a reversed repetition effect. In addition to the downstream effects of interest, we expected to observe a frontal positivity more immediately during prediction disconfirmation (e.g., Federmeier et al., 2007). Prediction disconfirmation could also be accompanied by a power increase in the theta band. Previous studies have observed theta increases in response to unexpected words or semantic anomalies compared with expected words (Bastiaansen & Hagoort, 2015; Hald et al., 2006; Rommers et al., 2017; Wang et al., 2012), which could reflect facilitated access of expected words, surprise about unexpected words, or both. To our knowledge, spectro-temporal responses to unexpected words in strongly constraining contexts have not previously been compared with a baseline of weakly constraining contexts, as we do here. Observing frontal positivity and/or theta effects would further allow for exploratory analyses of possible relationships between immediate and downstream effects of prediction disconfirmation.

## 2. Methods

### 2.1. Participants

Thirty-six native speakers of American English (23 women and 13 men; average age 21 years, range 18–31 years) gave informed consent and took part in the experiment in exchange for course credit or cash. The chosen sample size is six participants more than Rommers and Federmeier (2018) because a more subtle effect was expected a priori (no formal power analysis was conducted). All participants were right-handed (17 reported having left-handed family members) and had normal or corrected-to-normal vision. None reported a history of neurological or psychiatric disorders. Five additional participants were excluded; four because of EEG artifacts and one because of a technical error.

### 2.2. Materials and design

The experimental stimuli consisted of 123 sets of three sentences built around the same critical word (e.g., “hot”): two weakly constraining sentences with the critical word as their sentence-final completion (“He was surprised when he found out it was hot”, hereafter referred to as Weak Constraint Unexpected; and “The proofreader asked her to replace the word hot”, hereafter referred to as Critical Sentence), and one strongly constraining sentence from Federmeier et al. (2007) in which the critical word would have been the most predictable ending, but which instead ended in a plausible alternative word that had a low cloze probability and was semantically unrelated to the expected word (“Be careful, because the top of the stove is very dirty”, hereafter Strong Constraint Unexpected). As shown in Table 1, together with other intervening materials, different subsets of the three sentences were arranged to create three conditions, which always included the Critical Sentence, but differed in terms of what had been presented previously. In the Previously Seen condition, the critical word had been presented previously in the Weak Constraint Unexpected sentence. In the Expected But Not Seen condition, the critical word had been expected but not presented in a Strong Constraint Unexpected sentence. Finally, in the Not Previously Seen condition, the critical word had not been presented before.

The sentences had been selected from a larger set based on a sentence completion norming study (reported in Rommers and Federmeier, 2018). Following common practice, the cloze probability of a word in a sentence was operationalized as the proportion of an independent group of participants who completed the sentence with that word. The constraint of a sentence frame was operationalized as the cloze probability of its most frequent completion. The cloze probabilities of the two types of unexpected words were low (Weak Constraint Unexpected, mean ± SD: 0.01 ± 0.04, range 0–0.25, Strong Constraint Unexpected: 0.002 ± 0.01, range 0–0.10) and the length of the sentences in which they appeared was matched (Strong Constraint Unexpected: 10.02 ± 3.96 words, range 4–21; Weak Constraint Unexpected: 10.02 ± 3.95 words, range 4–21). Weak Constraint Unexpected sentences were less constraining (0.19 ± 0.08, range 0–0.35) than Strong Constraint Unexpected sentences (0.86 ± 0.13, range 0.45–1.00); in the latter, the most frequently provided completion was always the critical word. The critical sentences were 8.08 ± 2.23 words long (range 4–17 words), were weakly constraining (0.18 ± 0.08, range 0–0.35), and had low cloze probability sentence endings (0.01 ± 0.05, range 0–0.30). Critical words were rotated across the three conditions, so visual input was identical.

The sentences were divided across three presentation lists, on which each item occurred in only one condition (41 items per condition). The addition to each list of 82 fillers with moderately predictable sentence endings (average cloze probability 0.41, range 0.24–0.68) ensured that only 14% of the sentence endings constituted a repetition and that most sentence endings did not violate expectations. In each list, the 287 sentences were divided into 13 blocks of 21 sentences and one block of 14 sentences, pseudo-randomized individually for each participant. Two sentences intervened between the initial presentation/expectation of the critical word and the critical sentence, which always occurred in the same block. Because of randomization, the intervening sentences comprised fillers as well as Critical Sentences or Strong/Weak Constraint Unexpected sentences from other items.

### 2.3. Procedure

Participants were tested individually, seated 100 cm in front of a screen. They were asked to read the sentences for comprehension while avoiding blinks and eye and head movements. Stimuli were presented in white Arial size 20 font on a black background. On each trial, a central fixation cross appeared and remained on the screen for 650 ms, followed by a 350 ms blank screen. Then a sentence was presented word by word. Each word remained on the center of the screen for 200 ms, followed by a 300 ms blank screen. The blank screen after each sentence ending remained for 1500 ms, followed by three asterisks (* * *) for 2 s, which indicated the preferred time to blink. After each block, participants could take a break. After the reading task, they took an untimed paper-and-pencil recognition test as a measure of whether they had paid attention to the sentences. They were presented with an alphabetically ordered list of all 123 critical words and 123 new words similar in frequency and length and were asked to circle the words that they remembered reading. Finally, a verbal fluency test was administered in which participants produced as many words as they could in 1 min. In six versions of the task, they produced words beginning with a particular letter (“F”, “A”, “S”) or belonging to a particular semantic category (“animals”, “fruits and vegetables”, “first names”). Unrelated to the main goal of the study, this enabled further examination of a possible link between prediction and production (Dell and Chang, 2014; Federmeier, 2007; Pickering and Garrod, 2007). Responses were recorded and tallied on-line. We have reported all measures, conditions and data exclusions.

### 2.4. EEG recording and analysis

**Fig. 1.**
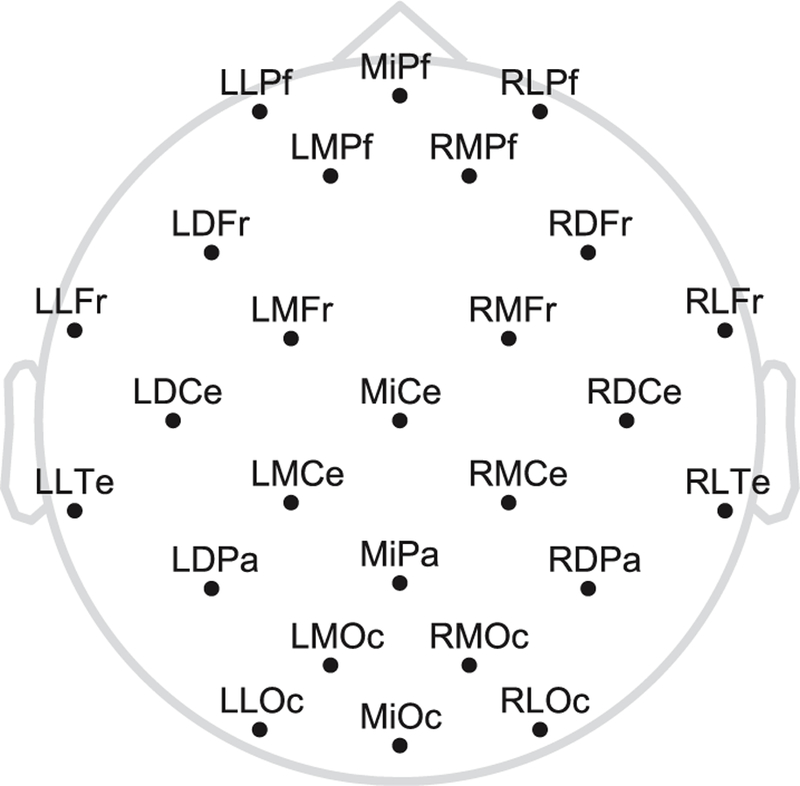
Schematic of the electrode montage with labels.

The EEG was recorded from 26 geodesically arranged Ag/AgCl electrodes mounted in a cap, referenced to the left mastoid (see Fig. 1). Additional electrodes were placed on the right mastoid, as well as on the left infraorbital ridge and on the outer canthus of each eye for electrooculogram (EOG) recordings. Electrode impedance was kept below 5 kΩ. The signal was amplified and digitized using BrainAmp amplifiers with a bandpass filter of 0.016–250 Hz and a sampling frequency of 1000 Hz.

The EEG was analyzed using EEGlab, ERPlab and Fieldtrip (Delorme and Makeig, 2004; Lopez-Calderon and Luck, 2014; Oostenveld et al., 2011). All analyses had the following preprocessing steps in common (and follow Rommers and Federmeier, 2018). The signal was re-referenced to the average of the left and right mastoids, high-pass filtered at 0.1 Hz (two-pass Butterworth with a slope of 12 dB/oct), and vertical and horizontal bipolar EOG derivations were calculated. The signal was then segmented into epochs spanning −750 to 1250 ms relative to critical word onset, and a 200 ms pre-stimulus baseline was subtracted. In four participants, 1–3 noisy channels were spline-interpolated. In the data of three participants with artifacts on more than 30% of the trials, trials with blinks (which predominantly occurred after the critical word had already been presented) were corrected using Adaptive Mixture Independent Component Analysis (AMICA; Palmer et al., 2011). Independent components that correlated with the vertical EOG at Pearson |*r|* > 0.60 were removed (one or two components per participant) and the corrected trials added back into the EEG record. Remaining trials containing blinks, eye movements, drifts, or excessive muscle activity were rejected using participant-specific thresholds. In total, 14.9% of the trials were rejected, with the following number of trials remaining in each condition (mean ± SD): Weak Constraint Unexpected 34 ± 4, Strong Constraint Unexpected 34 ± 3, Previously Seen 35 ± 3, Expected But Not Seen 35 ± 3, Not Previously Seen 35 ± 3.

#### 2.4.1. Event-related potentials

Trials were averaged point-by-point in the time domain for each participant and condition, and a 20 Hz low pass filter was applied (two-pass Butterworth with a slope of 24 dB/oct). To quantify the N400, mean amplitude measurements were taken in a 300–500 ms window, averaged across six centroparietal channels where the N400 tends to be maximal (LMCe, RMCe, MiCe, MiPa, LDPa, RDPa; following Wlotko et al., 2012). At initial presentation, the frontal positivity in response to the two types of unexpected words was measured as the mean amplitude in a 500–800 ms window across five frontal channels over each hemisphere (LLPf/RLPf, LMPf/RMPf, LDFr/RDFr, LMFr/RMFr, LLFr/RLFr) to allow for detecting left-lateralization seen in a previous study (Federmeier et al., 2007; see also the contrast against predictable words in DeLong et al., 2014; Kutas, 1993; Thornhill and Van Petten, 2012). For the repetition conditions, late positive complex (LPC) amplitude was measured in the same 500–800 ms window across the above-mentioned six parietal channels (e.g., Rugg et al., 1998). We further planned to explore relationships between effects observed at initial presentation/expectation and the downstream repetition effects at the trial level, using mixed-effects models which simultaneously take into account items and participants as random factors (Baayen et al., 2008).

#### 2.4.2. Time-frequency analysis of power

Time-frequency representations of power were calculated using a moving window Fast Fourier Transform (FFT) approach. A window of −500 ms moved along the time axis in 10 ms steps, centered from 500 to 1000 ms relative to critical word onset. Each instance of the window was Hanning-tapered and Fourier transformed, extracting frequencies from 4 to 30 Hz in 1 Hz steps (i.e., applying some interpolation). The resulting power spectrograms were averaged within each participant and condition, and normalized by dividing element-wise by the average power spectrogram across all conditions (rather than baseline correction, to avoid effects of pre-stimulus differences). In the absence of strong a priori knowledge about the nature of the repetition effects of interest, power differences between conditions during the 1 s after critical word onset were assessed across all frequencies, time points, and channels, using cluster-based permutation tests to control for multiple comparisons (Maris and Oostenveld, 2007). Statistically significant (*t*-test, *p* < .05) data points were clustered based on adjacency in time, frequency, or space (triangulation resulted in an average of 6.2 neighbors per channel), and the cluster with the largest summed *t* value was selected. This cluster-level *t* value was then compared with a benchmark distribution of *t* values obtained using the same procedure, but randomly permuting condition labels within participants 1000 times. The *p* value reflects the proportion of permutations in which the cluster-level *t* value was more extreme than the observed data.

## 3. Results

### 3.1. Behavioral memory performance

The percentage of words correctly recognized (43.3%) was higher by 28.0% (95% CI [23.9, 32.2], *d*_*z*_ *=* 2.28) than the percentage of false alarms to unseen words (15.3%). This difference was present in all participants and led to a by-participant average *d*’ of 0.936 (95% CI [0.800, 1.073]). Thus, participants successfully distinguished between seen and unseen words, suggesting that they had been paying attention to the sentences.

All of the previously seen words had been presented in a weakly constraining critical sentence, but some of them had also been presented in another weakly constraining sentence or had been expected but not presented in a strongly constraining sentence. Responses to these words (1 = judged seen, 0 = not judged seen) were analyzed using a logistic mixed effects model (Jaeger, 2008) with the fixed factor Prior Presentation (Seen Once, Seen Twice, Seen Once + Expected, treatment-coded), by-item random intercepts and random slopes for Prior Presentation and by-participant random intercepts (a model with by-participant random slopes did not converge, which would have been the maximal random effects structure warranted by the design; Barr et al., 2013). There was an effect of Prior Presentation, *χ*^*2*^ (2) = 41.261, *p* < .0001. Relative to words Seen Once (39.8%), words Seen Twice (50.5%) were recognized more often by 10.7% (95% CI [7.2, 14.2], *d*_*z*_ *=* 1.03), *β* = 0.517, *z =* 6.212, *p* < .0001. Words Seen Twice were also recognized more often than words Seen Once + Expected (39.7%), by 10.8% (95% CI [7.9, 13.7], *d*_*z*_ = 1.26), *β* = 0.518, *z* = 5.864, *p* < .0001. Recognition rates were similar for words Seen Once and words Seen Once + Expected (0.1% difference, 95% CI [−3.6, 3.7], *d*_*z*_ *=* 0.01), *β* = 0.001, *z =* 0.007, *p =* .9946. Thus, repetition increased the probability of recognition, but having expected a word prior to having seen it did not affect memory judgments at the end of the experiment.

### 3.2. Event-related potentials

**Fig. 2.**
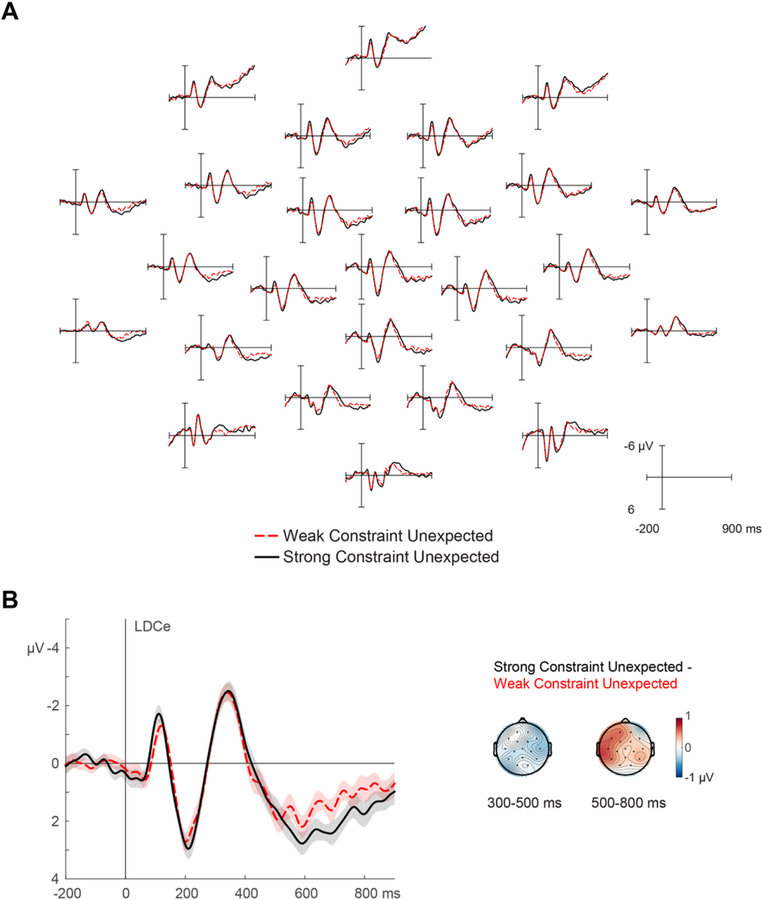
Grand-average ERPs time-locked to words upon initial presentation. Words disconfirmed a likely expectation (induced by a strongly constraining sentence context) or were generally unexpected (presented in a weakly constraining sentence context). Negative is plotted up in all ERP figures. A) All scalp electrode sites; the position of the channels in the figure approximates the position on the head, with the nose at the top. B) Close-up of a left-frontocentral channel (LDCe) showing the frontal positivity. Shading reflects unbiased within-subjects SEM (Cousineau, 2005; Morey, 2008). Insets show scalp topographies of the N400 and frontal positivity difference wave (Strong Constraint Unexpected – Weak Constraint Unexpected).

ERPs elicited at initial presentation are shown in Fig. 2. Confirming previous studies, after the visual N1 and P2, the N400 in response to the two types of unexpected words was relatively unaffected by sentential constraint (differing numerically by 0.26 μV, 95% CI [−0.45, 0.96], *d*_*z*_ *=* 0.12), *F* (1,35) = 0.5387, *p =* .4679.^3^ After the N400, amplitudes over frontal channels showed a Constraint × Hemisphere interaction of 0.51 μV (95% CI [0.11, 0.90], *d*_*z*_ *=* 0.43), *F* (1,35) = 6.7745, *p =* .0135. Similarly to previous studies, this reflected a frontal positivity in strongly constraining relative to weakly constraining contexts, here occurring on channels over the left hemisphere (0.56 μV, 95% CI [0.14, 0.98], *d*_*z*_ = 0.46), *F* (1,35) = 7.4768, *p* = .0097, but not over the right (0.05 μV, 95% CI [−0.39, 0.50], *d*_*z*_ *=* 0.04), *F* (1,35) = 0.0576, *p =* .8117. Participants with greater semantic verbal fluency scores showed a larger left frontal positivity effect, *r =* 0.36, *p =* .0322, extending previous findings linking electrophysiological indices of prediction to production (Federmeier et al., 2002, 2010; cf. Wlotko et al., 2012). In sum, ERP responses at initial presentation demonstrated sensitivity to prediction disconfirmation.

**Fig. 3.**
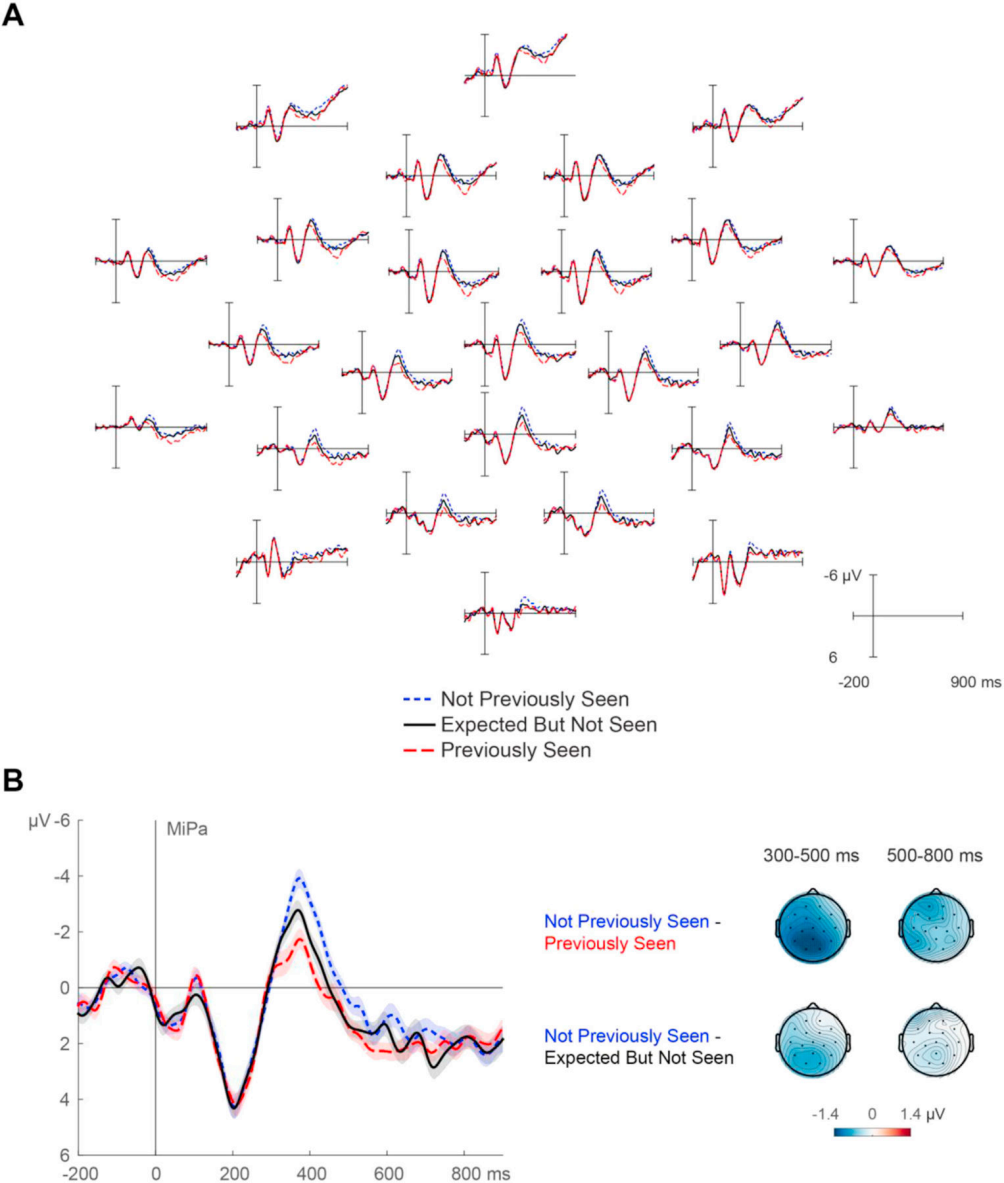
Grand-average ERPs time-locked to sentence-final words in the critical weakly constraining sentences. The words were either repetitions (Previously Predictable, Previously Unpredictable) or unseen words presented in the same sentence contexts (Not Previously Seen). A) All scalp electrode sites. B) Close-up of a centro-parietal channel (MiPa). Shading reflects unbiased within-subjects SEM (Cousineau, 2005; Morey, 2008). Scalp topographies show the repetition effects for previously unpredictable words and for expected but not seen words.

ERPs elicited by the critical sentence endings are shown in Fig. 3. The N400 differed between the three conditions, *F* (2,70) = 11.444, *p =* .0001 (Greenhouse-Geisser corrected, *ε =* 0.8792). As expected, relative to Not Previously Seen words, Previously Seen words attenuated the N400 (a repetition effect) by 1.30 μV (95% CI [0.81, 1.79], *d*_*z*_ = 0.89), *F* (1,35) = 28.7567, *p* < .0001. Critically, Expected But Not Seen words also attenuated the N400, by 0.70 μV (95% CI [0.19, 1.20], *d*_*z*_ *=* 0.47), *F* (1,35) = 7.8110, *p =* .0084. The N400 reduction in response to Previously Seen words was larger than that in response to Expected But Not Seen words by 0.61 μV (95% CI [−0.04, 1.25], *d*_*z*_ *=* 0.32), *F* (1,35) = 3.6162, *p =* .0655.

Following the N400, the LPC also differed between conditions, *F* (2,70) = 2.6793, *p =* .0848 (Greenhouse-Geisser corrected, *ε* = 0.8561). Replicating earlier word repetition studies, relative to Not Previously Seen words, the LPC in response to Previously Seen words was enhanced by 0.68 μV (95% CI [0.17, 1.19], *d*_*z*_ *=* 0.45), *F* (1,35) = 7.3946, *p =* .0101. The LPC in response to Expected But Not Seen words was of intermediate amplitude, differing only numerically from the Not Previously Seen words (by 0.31 μV, 95% CI [−0.40, 1.02], *d*_*z*_ = 0.15), *F* (1,35) = 0.7838, *p* = .3820, and from the Previously Seen words (by 0.37 μV, 95% CI [−0.19, 0.93], *d*_*z*_ *=* 0.22), *F* (1,35) = 1.8207, *p* .1859. In sum, the N400 was reduced in response to repeated or merely expected words, whereas the LPC was only notably enhanced in response to actually repeated words.

Further analyses examined whether, and if so, how, the N400 elicited by previously seen and previously expected words was related to the frontal positivity in response to unexpected words at initial presentation. This question was examined in the subset of items for which both the first presentation/expectation and the corresponding critical sentence had passed artifact rejection (trial number average ± SD: Previously Predictable 30 ± 4; Previously Unpredictable 30 ± 4). The Not Previously Seen condition was left out of these analyses, because it did not have a corresponding initial presentation/expectation sentence to use as a predictor. Trial-level mean amplitude of the N400 in the critical sentences (across the 300–500 ms time window at the six centroparietal channels mentioned above) was predicted on the basis of Prior Presentation condition and Prior Frontal Positivity Amplitude (across a 500–800 ms time window at the 5 left frontal channels, z-scored relative to each participant’s condition average) using a linear mixed-effects regression model. However, Prior Frontal Positivity yielded no main effect, *β* =−0.047, *t* =−0.274, *χ*^*2*^ (1) = 0.0736, *p =* .7861, nor was there a simple effect of Prior Frontal Positivity Amplitude at either level of Prior Presentation (Previously Seen: *β* = 0.316, *t* = 1.256, *p* = .2103; Expected But Not Seen: *β* = −0.411, *t* = −1.638, *p* = .1032).^4^

### 3.3. Time-frequency analysis

**Fig. 4.**
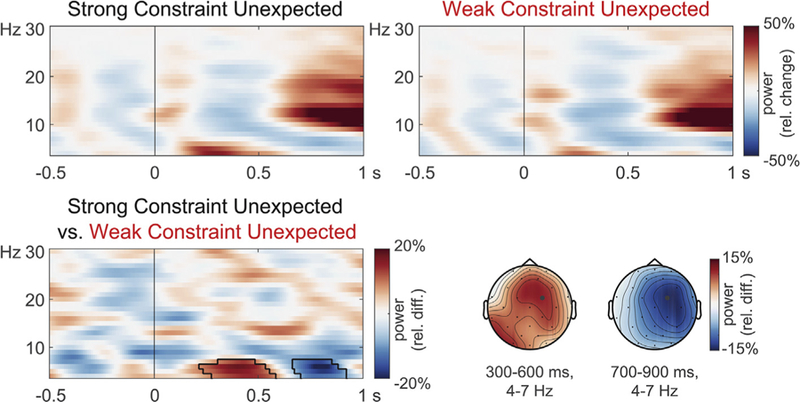
Grand-average time-frequency representations of power time-locked to word onset at initial presentation at a right frontocentral channel (RMFr; indicated with a black dot in the scalp maps). Spectrograms of individual conditions (relative to a –500 to –150 ms baseline) and their difference (relative to the average across all conditions) are shown along with scalp topographies of the differences. The contour lines in the spectrogram indicate cluster extent in permutation tests of the theta band difference.

Power changes time-locked to the two types of unexpected word at initial presentation are shown in Fig. 4. Relative to a pre-stimulus baseline, the words elicited early short-lived power increases followed by a broadly distributed theta increase and an alpha/beta decrease with frontal and occipital maxima, and a late broadly distributed alpha/beta increase and late frontal/occipital theta decrease. Power tended to differ between the two types of unexpected words, *p =* .0899, with a cluster suggesting a late theta decrease. An analysis within the theta band (4–7 Hz), based on its previously reported association with unexpected words (Hald et al., 2006; Rommers et al., 2017; Wang et al., 2012), confirmed the power increase visible between 300 and 600 ms, *p =* .0100, as well as the power decrease around 700–900 ms, *p =* .0200.^5^ A mixed-effects model in which trial-level theta power in these time windows served as predictors did not reveal a relationship between these neural signatures and N400 amplitude elicited by the critical word further downstream, ts < 1.341, ps > .1802.

**Fig. 5.**
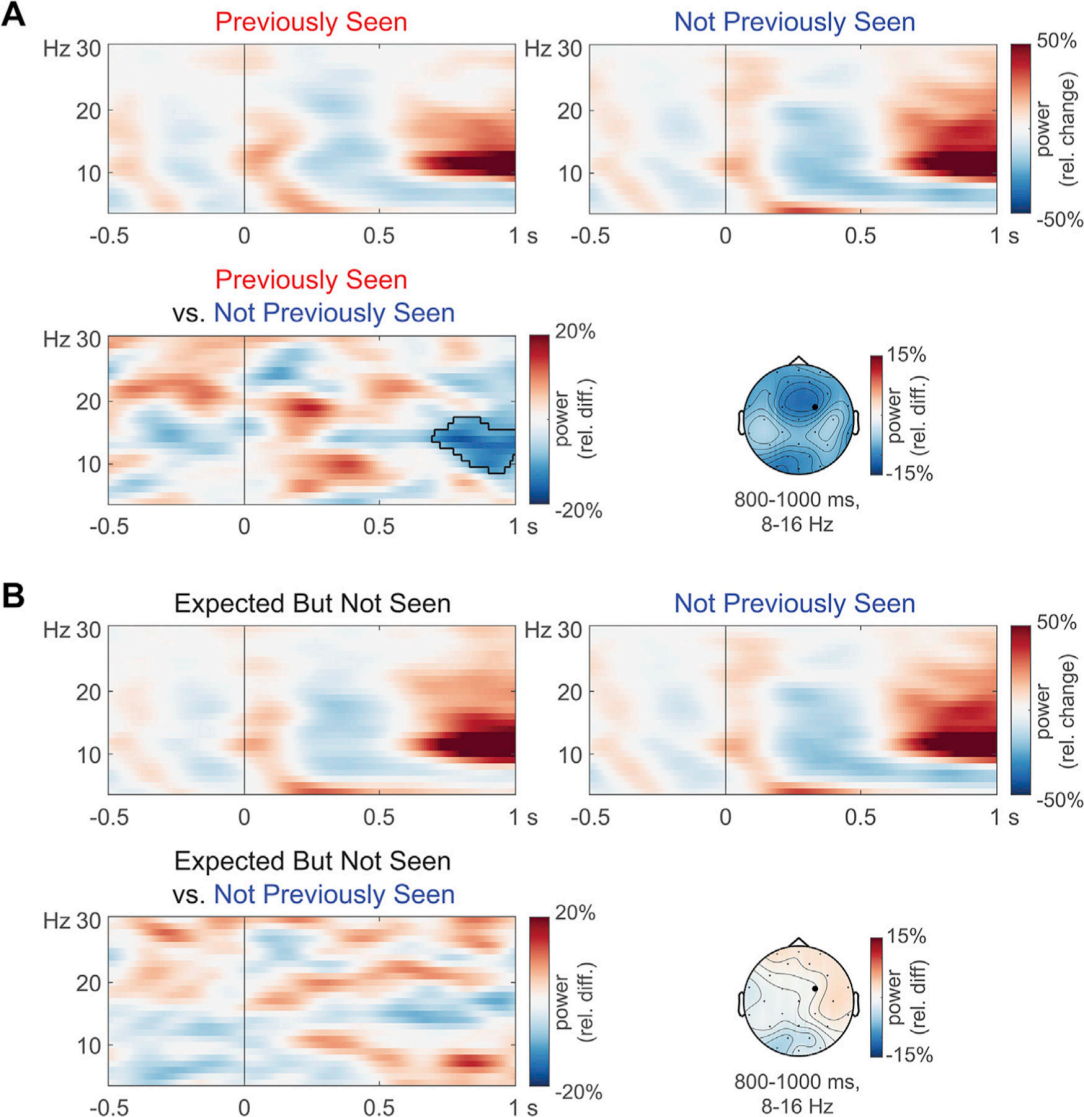
Grand-average time frequency representations of power time-locked to final words in the critical sentences, at a right frontocentral channel (RMFr; indicated with a black dot in the scalp maps). The top pairs of spectrograms within each panel show power changes relative to a −500 to −150 ms baseline within each condition. The bottom spectrograms show power differences (relative to the average across all conditions) and their scalp topography. Contour lines indicate cluster extent in permutation tests. A) Repetition effect. B) No pseudo-repetition effect for expected but not presented words.

Power changes time-locked to the critical sentence endings are shown in Fig. 5. Relative to Not Previously Seen words, Previously Seen words elicited a late alpha/beta power decrease around 800–1000 ms over frontal and occipital channels (visual inspection suggested that the part of the cluster that extended into the beta frequencies was the main contributor to the frontal decrease). This repetition effect was detected as a cluster, *p =* .0340. For Expected But Not Seen words, no such difference was observed, *p =* .9850. The alpha decrease in response to Previously Seen words was also visible relative to Expected But Not Seen words, but statistically not detected, *p =* .1598. For illustration only, we inspected the size of the effects where they were observed, averaged between 800 and 1000 ms and 8–16 Hz across five frontocentral channels (LMPf, RMPf, LMFr, RMFr, MiCe). The basic repetition effect was −9.7% (95% CI [−4.3, −15.2], *d*_*z*_ *=* 0.60), the difference between Not Previously Seen and Expected But Not Seen words was 0.6% (95% CI [−5.3, 6.5], *d*_*z*_ = 0.03), and the difference between Previously Seen and Expected But Not Seen words was −10.3% (95% CI [−3.7, −16.9], *d*_*z*_ *=* 0.53). In sum, late alpha/beta power was only reduced in response to actual repetition.

## 4. Discussion

Previous studies have identified electrophysiological signatures of prediction disconfirmation during rapid on-line language processing, without addressing whether prediction has consequences further downstream. In particular, it is unclear what happens with expected information if it has been disconfirmed: is it suppressed, or does it linger? This study manipulated whether words were actually seen or were only expected – but disconfirmed – and then probed their fate in memory by presenting the words (again) a few sentences later.

At initial presentation, relative to words that were unexpected because they appeared in weakly constraining contexts, unexpected words in strongly constraining sentence contexts, where they constituted prediction violations, elicited a left-lateralized frontal positivity and a frontal theta increase. These effects may be related and may reflect aspects of dealing with disconfirmed expectations (e.g., Federmeier et al., 2007; Rommers et al., 2017). In addition, the frontal theta increase was followed by a theta decrease, which has not previously been observed in response to word prediction disconfirmation and we therefore refrain from interpreting it. It should also be kept in mind that the stimuli in this comparison were not identical (a consequence of optimizing the counterbalancing for the critical sentences). Most critically, the fact that there were effects of prediction disconfirmation suggests that participants had formed expectations.

Our main interest was in the repetition effects further downstream. Replicating prior work, repeated words, relative to previously unseen ones, elicited a strong N400 decrease (e.g., Rugg, 1985; Van Petten et al., 1991), an enhanced LPC (e.g., Besson et al., 1992; Rugg et al., 1998), and late alpha band power decreases (e.g., Rommers and Federmeier, 2018; Van Strien et al., 2007). Strikingly, like repeated words, expected but not seen words also attenuated the N400. This suggests that, despite having been disconfirmed, previously expected information remained relatively accessible in memory. Whereas an earlier behavioral study seemed to indicate a lack of lingering in young adults (Hartman and Hasher, 1991), the “pseudo-repetition effect” observed here shows that disconfirmed expectations are not fully suppressed and can linger. This finding is consistent with earlier evidence for lingering representations of actually presented input (e.g., Christianson et al., 2001) or likely expected parses (Kaschak and Glenberg, 2004), and extends it to merely expected words.

One may argue that the pseudo-repetition effect could reflect associative priming by words from the prior, expectation-disconfirming sentence (for example, “stove” priming “hot”). The present study was not designed to rule this out. In our view, distinguishing priming from prediction is not trivial, given that expectation (prediction) has been implicated as one source of associative priming effects (e.g., Neely, 1991) and that prediction during language processing likely encompasses a range of processes, including some that may be akin to spreading activation (e.g., Kuperberg and Jaeger, 2016). However, if one wanted to try to distinguish these, one might define simple associative priming as facilitation due to passive spreading of activation, which typically strongly decreases or dissipates after a few intervening words (e.g., Simpson et al., 1989; Van Petten et al., 1997). In this case, the label “prediction” seems to better describe our findings, because prediction has been theorized to have long-lasting effects (e.g., Chang et al., 2006).

The pseudo-repetition effect on the N400 was not as strong as the regular repetition effect. This may be simply because merely expecting a word does not result in the same amount of semantic processing as actually seeing it. However, in recent work that used the same repetition paradigm but actually presented the predictable word (Rommers and Federmeier, 2018), we found that one consequence of predictability is to reduce downstream repetition effects for the predictable word (presumably because readers did not encode the stimulus as thoroughly). The similarity in the size of the repetition effect across these two studies raises an intriguing question for future work, namely whether, at some stages of processing, the fate of predictable words in memory is similar whether they are actually presented or not. Future studies could also look into item factors that may influence whether lingering is observed: for instance, to what extent an unexpected word ‘negated’ a prediction (our stimuli seem to represent a mix that is difficult to classify).

A later facet of the repetition effect, the LPC, did not show a pseudo-repetition effect. This suggests that lingering of disconfirmed expectations resulted in priming, but not in explicit (false) recollection. Power in the alpha/beta band showed a similar pattern: a decrease in response to repeated words, but not in response to previously expected but not presented words. These power decreases may release task-relevant brain areas from ongoing inhibition (Jensen and Mazaheri, 2010; Klimesch et al., 1997) in the service of re-activating memory traces (Klimesch et al., 2005). The present study highlights the multifaceted nature of the repetition effect because, unlike the N400, these power decreases patterned with veridical memory. The lack of a pseudo-repetition effect on EEG indices of explicit recognition is consistent with the results from the recognition test performed at the end of the experiment: relative to having seen a word once, repetition increased the likelihood of recognizing a word, but merely having expected a word did not.

In summary, at the level of semantic processing, it appears that the brain does not consistently or completely suppress expectations for likely upcoming input when those expectations turn out to be incorrect. This result reveals suboptimal performance if the goal is to create a veridical representation of the input. At the same time, this failure to suppress expected information based on a single disconfirmation might form part of adaptive behavior. Particularly in realistic situations with noisy or incomplete input, it may be helpful to maintain or keep accessible an expectation that is more often correct than incorrect (see also Bicknell et al., 2016). The utility or degree of suppression could depend on the relative weights of the input and prior experience (as governed by, for example, a currently unknown learning rate), an area ripe for further research (see also Chang et al., 2006; Jaeger and Snider, 2013). Overall, the results demonstrate that prediction has consequences beyond rapid on-line processing.

## Figures and Tables

**Table 1:** Examples of the stimuli.

Previously Seen	
Weak Constraint Unexpected	He was surprised when he found out that it was hot.
Filler	The mother of the tall guard had the same accent.
Filler	The lawyer feared that his client was guilty.
Critical sentence	The proofreader asked her to replace the word hot.

**Expected But Not Seen**	

Strong Constraint Unexpected	Be careful, because the top of the stove is very dirty.
Filler	The mother of the tall guard had the same accent.
Filler	The lawyer feared that his client was guilty.
Critical sentence	The proofreader asked her to replace the word hot.

**Not Previously Seen**	

Filler	The final score of the game was tied.
Filler	The mother of the tall guard had the same accent.
Filler	The lawyer feared that his client was guilty.
Critical sentence	The proofreader asked her to replace the word hot.

*Note.* Critical words are underlined. The critical sentence was always weakly constraining, but the conditions differed in terms of what participants had previously seen. Because of randomization, in the actual experiment the intervening sentences (shown as Filler here for clarity) could be any part of the materials.

## Footnotes

1 Throughout the paper, the terms “theta power” or “alpha power” are used descriptively as shorthands for power changes that include the 4–7 Hz or 8–12 Hz range, thus remaining agnostic with respect to their possible physiological interpretation as theta/alpha oscillations.

2 In addition, work on speech disfluencies has shown that language-mediated anticipatory eye movements to objects can linger to some extent, despite a speaker’s repair canceling the predictive cue on which the expectation was based (Corley, 2010). On the other hand, after naming a predictable picture in a sentence context, other possible sentence completions do not seem to linger (Kleinman et al., 2015).

3 One may wonder whether, just prior to the frontal positivity, ERPs over occipital channels were more negative-going in response to the Strong Constraint Unexpected words than the Weak Constraint Unexpected words. Averaged across occipital channels (LMOc, RMOc, LLOc, RLOc, MiOc) and a 400–600 ms time window, the difference was 0.72 μV (*SE* = 0.31, *d*_*z*_ = 0.38). Because this unexpected finding was prompted by visual inspection, we sought to replicate it in another dataset (Federmeier et al., 2007) and observed a similar difference of 0.52 μV (95% CI [0.05, 0.99], *d*_*z*_ = 0.40), *F*(1,31) = 5.163, p = .0301. More data are necessary to allow for interpretation, but we note that a few other studies have reported or theorized about a late N400-like effect (Baggio and Hagoort, 2011; Brothers et al., 2015).

4 A reviewer pointed out that the manipulation employed in this study created differences between conditions in terms of the experimental environment in which the sentences occurred. Recent work suggests that adaptations to such differences in experimental environment can have a general effect on the N400 that subsequent input elicits (Delaney-Busch et al., 2017). Specifically, in the Expected But Not Seen condition, the cloze probability of the final word three sentences ago was low; in the Not Previously Seen condition, the cloze probability three sentences ago depended on randomization: it was low if an experimental sentence was presented, but moderate-to-high in case a filler was presented. Although future research could investigate this experimentally, an analysis within the Not Previously Seen condition revealed no clear evidence that moderate cloze fillers three sentences ago made the downstream N400 more negative than low cloze experimental sentences did, *β* = −0.51, *t* = −1.093, *χ*^*2*^ = 1.1808, *p* = .277.

5 On reviewer request, we additionally explored lower frequencies (i.e., 2–3 Hz) and higher frequencies (30–100 Hz). These analyses suggested that the power increase continued at lower frequencies (though note that the 500 ms window likely has low frequency precision in this range). In the higher frequencies, analyses using multitapers revealed no clear effects for any comparison (all cluster *p* > .3457).
